# Improved knee biomechanics among patients reporting a good outcome in knee-related quality of life one year after total knee arthroplasty

**DOI:** 10.1186/s12891-017-1479-3

**Published:** 2017-03-21

**Authors:** Josefine E. Naili, Per Wretenberg, Viktor Lindgren, Maura D. Iversen, Margareta Hedström, Eva W. Broström

**Affiliations:** 1 Department of Women’s and Children’s Health, Karolinska Institutet, MotorikLab, Q2:07, Karolinska University Hospital, 171 76 Stockholm, Sweden; 2 Department of Orthopedics, School of Medical Sciences, Örebro University and Örebro University Hospital, Örebro, Sweden; 3 Department of Molecular Medicine and Surgery, Karolinska Institutet, L1:00, Karolinska University Hospital, 171 76 Stockholm, Sweden; 4 Department of Physical Therapy, Movement & Rehabilitation Sciences, Bouve College of Health Sciences, Northeastern University, 360 Huntington Avenue, Boston, MA 02115 USA; 5 Division of Rheumatology, Immunology, and Allergy, Brigham and Women’s Hospital, Harvard Medical School, Boston, MA USA; 6 Department of Clinical Science, Intervention and Technology, Karolinska Institutet, Karolinska University Hospital, K54, 141 86 Stockholm, Sweden

**Keywords:** Gait, Knee, Biomechanics, Joint replacement, Quality of Life, Function, Osteoarthritis

## Abstract

**Background:**

It is not well understood why one in five patients report poor outcomes following knee arthroplasty. This study evaluated changes in knee biomechanics, and perceived pain among patients reporting either a good or a poor outcome in knee-related quality of life after total knee arthroplasty.

**Methods:**

Twenty-eight patients (mean age 66 (SD 7) years) were included in this prospective study. Within one month of knee arthroplasty and one year after surgery, patients underwent three-dimensional (3D) gait analysis, completed the Knee Injury and Osteoarthritis Outcome Score (KOOS), and rated perceived pain using a visual analogue scale. A “good outcome” was defined as a change greater than the minimally detectable change in the KOOS knee-related quality of life, and a “poor outcome” was defined as change below the minimally detectable change. Nineteen patients (68%) were classified as having a good outcome. Groups were analyzed separately and knee biomechanics were compared using a two-way repeated measures ANOVA. Differences in pain between groups were evaluated using Mann Whitney *U* test.

**Results:**

Patients classified as having a good outcome improved significantly in most knee gait biomechanical outcomes including increased knee flexion-extension range, reduced peak varus angle, increased peak flexion moment, and reduced peak valgus moment. The good outcome group also displayed a significant increase in walking speed, a reduction (normalization) of stance phase duration (% of gait cycle) and increased passive knee extension. Whereas, the only change in knee biomechanics, one year after surgery, for patients classified as having a poor outcome was a significant reduction in peak varus angle. No differences in pain postoperatively were found between groups.

**Conclusion:**

Patients reporting a good outcome in knee-related quality of life improved in knee biomechanics during gait, while patients reporting a poor outcome, despite similar reduction in pain, remained unchanged in knee biomechanics one year after total knee arthroplasty. With regards to surgeon-controlled biomechanical factors, surgery may most successfully address frontal plane knee alignment. However, achieving a good outcome in patient-reported knee-related quality of life may be related to dynamic improvements in the sagittal plane.

## Background

The majority of patients with knee osteoarthritis report decreased pain and improved function following total knee arthroplasty (TKA), yet nearly one in five report limited function, persistent disability, and reduced quality of life (QoL) [1–3]. The reasons for persistent disability in this subset of patients are not well understood [1]. Patient satisfaction after TKA is associated with patient-reported outcomes and clinician assessments [4], although surgeons tend to report greater satisfaction with surgical results than patients [5]. In a study investigating factors associated with patient satisfaction after TKA, the authors found no differences between satisfied and dissatisfied patients with regards to clinical examination findings, performance-based function, and radiography, although perceived knee pain differed between the groups, wherein satisfied patients reported lower postoperative pain levels [6].

Patient-reported outcomes are frequently used for evaluating pain and function following TKA. The Knee Injury and Osteoarthritis Outcome Score (KOOS) is one of few disease-specific instruments for patients with knee osteoarthritis that includes a subscale measuring knee-related QoL. The knee-related QoL subscale evaluates knee-specific mental and social aspects of function and requires patients to reflect upon the impact of knee symptoms on their QoL [7]. This subscale may be considered the most emotionally sensitive part of the questionnaire as it evaluates awareness and lifestyle changes related to the knee [7]. The knee-related QoL subscale is the most responsive KOOS subscale when outcomes are measured at six and 12 months after TKA [7]. Additionally, 90% of patients with TKA consider this subscale to be extremely or very important [8].

At one year follow-up, studies using three dimensional (3D) gait analysis to evaluate outcomes after TKA have shown that knee biomechanics and gait pattern do not return to normal after surgery [9–12]. Knee kinematics and kinetics during gait continue to deviate compared to healthy controls, as represented by reduced knee flexion-extension range, reduced peak flexion moments, and increased external knee adduction moments [9, 10]. The frequency and severity of anterior knee pain after TKA appear to partially be explained by a retained higher preoperative external knee flexion moment [13]. To the best of our knowledge, no prior studies have evaluated change in knee biomechanics during gait in patients grouped according to their postoperative self-reported outcome. Therefore, the primary aim of this study was to evaluate change in knee biomechanics during gait among patients reporting a good or a poor outcome in knee-related QoL, respectively. Secondly, we wanted to evaluate if the good or poor outcome groups reported differences in perceived pain postoperatively.

## Methods

### Participants

Between the years 2010 and 2014, 40 patients were recruited from two orthopedic departments in Stockholm County, Sweden (Ortho Center, Löweströmska Hospital and the Department of Orthopedics at Karolinska University Hospital). Inclusion criteria were: being scheduled for TKA within one month due to primary knee osteoarthritis, the ability to walk 10 m repeatedly without assistance of a walking aid, and the ability to understand verbal and written information in Swedish. Exclusion criteria were: other severe joint pain or previous major orthopedic surgery in the lower extremities (including traumatic knee injury), rheumatoid arthritis, neurological disorder, diabetes mellitus, body mass index (BMI) > 40, and/or other medical condition affecting walking ability. All patients that met inclusion and exclusion criteria and accepted participation were included in this prospective cohort study [11]. Twenty-five age and gender matched, healthy controls without any known musculoskeletal disease or neurological disorder were recruited through acquaintances between the years 2013–2015. The control group was matched to the osteoarthritis group by age strata across five age groups (40–49, 50–59, 60–69, 70–79, 80–89 years of age). The mean age of the control group was 66 years (SD 10), and they had a mean BMI of 24.9 (SD 2.9). The present study is a secondary analysis and therefore, participants and methods are described more in detail elsewhere [11]. The study was approved by Stockholm’s regional ethical review board (DNR: 2010/1014-31/1), and all study participants provided written informed consent in accordance with the Declaration of Helsinki.

Out of the 40 patients included at baseline, 28 patients (18 females), with a mean age of 66 (SD 7) years, completed the one year follow-up. Reasons for not completing the one year follow up were: not going through with the planned surgery (*n* = 2), post-operative infection causing re-operation (*n* = 2), TKA in the contralateral limb within the following year (*n* = 5), death (*n* = 1), pelvic fracture during the following year (*n* = 1), and cancer diagnosis (*n* = 1). Patients who did not complete the one year follow up (*n* = 12) did not differ statistically from the studied group with regards to distribution of age, gender, weight, height, BMI or duration of years with symptomatic knee osteoarthritis.

### Setting and procedures

Baseline evaluations were performed within one month prior to surgery (mean 20 (SD 13) days) and postoperative evaluations one year after surgery (mean 12 (SD 0.9) months). Three-dimensional gait data and patient-reported outcomes were collected at the Motion Analysis Laboratory at Karolinska University Hospital, Solna, Sweden. Each test session started with a physical examination. Passive range of motion of the lower extremity joints were recorded using a goniometer with the patient in a supine position for all measures except hip extension which was recorded with the patient in a prone position. Anthropometric measures were recorded using calibrated scales. After the initial examination, 35 retro-reflective markers were placed on anatomical landmarks (head, trunk, pelvis, lower and upper extremities), according to the conventional biomechanical model Plug-In-Gait (Vicon Motion Systems Ltd, Oxford, UK) [14].

### Three-dimensional gait analysis

Three-dimensional gait data were collected using an eight camera system (Vicon Motion Systems Ltd, Oxford, UK) (sampling rate 100 Hz), and two force plates embedded in the floor (Kistler, Winterthur, Switzerland) (1000 Hz). Kinematic, kinetic, and time and distance parameters were collected simultaneously. Kinetics were expressed as internal moments (forces from muscles, ligaments and tendons acting on the specific joint). The studied kinematic variables included knee flexion-extension range during an entire gait cycle, peak knee flexion angle in swing phase, and peak varus angle during stance phase. The studied kinetic variables during stance phase included peak knee flexion moment, peak knee extension moment, and peak knee valgus moment. Patients were instructed to walk barefoot at self-selected speed along a defined 10 m walkway. Recordings were made in two directions (back and forth). Approximately five gait trials, with clean force plate strikes, were analyzed for each patient at each test session. Gait variables from these strides were averaged to obtain one value for each variable of interest, for each patient, at each test session. Raw motion capture data were filtered in a Woltring Filter [15] with a mean squared error setting of 15, and 3D gait kinematics and kinetics were computed according to the Plug-in-Gait model [14]. The gait kinematics and kinetics data were then exported to the software program MATLAB®, R2014a (The MathWorks, Inc, Natick, MA, USA) where discrete values (maxima, minima) were computed for the participants.

### Perceived pain

After completing the gait trials, patients rated their perceived pain experienced during the gait trials using a visual analogue scale (VAS) where 0 represents “no pain” and 100 mm represents “severe pain” [16].

### Patient-reported outcomes

The KOOS was used to evaluate patient-reported pain, symptoms, function in activities of daily living (ADL), function in sport and recreation, and knee-related QoL [17]. KOOS generates a final score for each separate subscale, ranging from 0-100, where 0 represent “severe difficulties” and 100 “no problems at all”. Adequate test-retest reliability (intra class correlation range 0.85 – 0.9) has been reported for all subscales [18]. The questionnaire is widely used, and is valid and responsive to change in patients with knee osteoarthritis receiving both conservative [19] and surgical treatment [20].

### Radiological classification

According to standard practice at each hospital, preoperative standing anterior-posterior radiographs were taken. Two experienced orthopedic surgeons together performed the classification of radiographic severity of osteoarthritis (for the knee joint as a whole) according to the modified Kellgren and Lawrence’s (KL) classification [21] (Table 1).

**Table 1:** Baseline characteristics, within one month prior to knee arthroplasty. Patients are grouped according to postoperative change in knee-related Quality of Life

	Good outcome group, *n* = 19	Poor outcome group, *n* = 9	Difference between groups, *p*-value
Female, n	12	6	0.856
Age, years, mean (SD)	67.3 (7.7)	62.4 (5.4)	0.1
Height (cm), median (range)	170 (156-184)	163 (159-181)	0.438
Weight (kg), mean (SD)	82.8 (11.8)	83.5 (14.3)	0.892
BMI (m2/kg), mean (SD)	29 (4.6)	30 (5.1)	0.614
Symptom duration, years, median (range)	10 (1.5-26)	8 (1-20)	0.263
Kellgren and Lawrence score (1-4b)			
1-2	-	-	
3a, n	0	1	0.321
3b, n	1	3	0.084
4a, n	7	1	0.214
4b, n	11	4	0.689
Knee Injury and Osteoarthritis Outcome Score, 0-100			
Pain, mean (SD)	48 (14)	40 (17)	0.218
Symptoms, median (range)	32 (18-89)	32 (18-75)	0.699
Activities of Daily Living, mean (SD)	62 (11)	46 (19)	*0.007*
Sport and Recreation, median (range)	15 (0-60)	15 (0-80)	0.809
Knee-related Quality of Life, mean (SD)	30 (13)	24 (9)	0.251

Poor outcome was defined as change below the minimally detectable change (21.1) in knee-related Quality of Life of KOOS

Italicized *p*-value indicating a significant difference (*p*<0.05)

### Knee replacement surgery and postoperative rehabilitation

The surgical procedures were performed by seven different senior orthopedic surgeons from two hospitals, all using a posterior cruciate ligament retaining cemented TKA (PFC-Sigma, DePuy, Johnson & Johnson, Warsaw, Poland). Surgeons were equally distributed across the two groups. After surgery, patients were allowed full weight bearing (together with use of an appropriate walking aid), and unrestricted range of motion. The postoperative rehabilitation was performed according to the standard practice at each hospital (in-patient physiotherapy <1 week). Thereafter, patients received rehabilitation in a primary care setting of their choice, which lasted for a median duration of 3 months (range 1-6 months) in the good outcome group and 5 months (range 1-6 months) in the poor outcome group.

### Classification of outcome

**Fig. 1:**
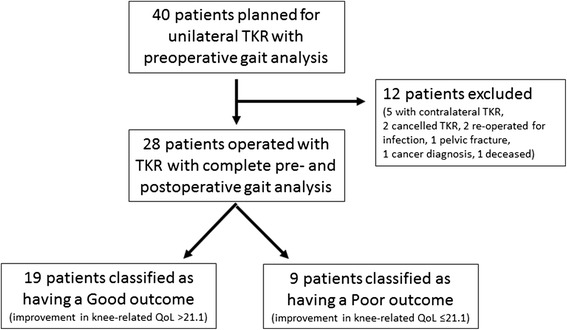
Flowchart of included patients with knee osteoarthritis scheduled for total knee arthroplasty (TKA), excluded patients and patients with complete pre- and postoperative assessments. Patients are grouped according to postoperative change in knee-related Quality of Life (QoL)

The minimally detectable change (MDC) of KOOS knee-related QoL is reported to be 21.1 points one year after TKA [8, 22], and this was used as a cut-off to classify individuals postoperative outcome as either a “good outcome” (change ≥ MDC), or a “poor outcome” (change < MDC). The MDC is a statistical estimate that provides a threshold for interpretation of a measurement [23]. When a change score exceeds this threshold, there is reasonable certainty that it represents a true change, and not a measurement error [23]. The MDC is not an absolute value, and should be considered a guideline [23]. In this sample, 19 (68%) out of 28 patients reported change greater than the MDC in KOOS knee-related QoL at the one year follow-up, and were classified as having a good outcome (Fig. 1). Nine patients reported change smaller than 21.1 points in knee-related QoL and were classified as having a poor outcome. The good and poor outcome groups were analyzed separately and compared (Table 1).

### Statistical analysis

A significance level was set at p <0.05 and data analyses were performed using IBM SPSS Statistics version 22 (Chicago, IL, USA). Depending on data distribution, means with standard deviation and medians with range, were used to describe the explored variables. Normal distribution of data was assessed using Shapiro-Wilk’s test and Q-Q plots. Sample size calculations were made a priori for the primary analysis with another aim [11]. In this secondary analysis, post hoc power analyses were performed with regards to change in knee gait biomechanics. The sample size of the good outcome group (*n* = 19) had sufficient power (range 93-99%) to detect significant differences in knee flexion-extension range, peak varus angle and peak valgus moment within the group [24]. Power for the corresponding variables within the poor outcome group (*n* = 9) was low (range 23-55%) [24]. Knee gait biomechanics, passive range of knee motion, and time and distance parameters were analyzed using a two-way repeated measures ANOVA with the within groups factor Time (prior to TKA and one year post TKA) and the between groups factor Group (the good outcome group and the poor outcome group) and the interaction Group*Time. The Group*Time interaction refers to the statistical test of whether the response profile for one group is the same as for the other group. In case of a significant interaction, simple effects were tested, i.e. effects of one factor holding the levels of the other factor fixed. To adjust for preoperative differences between groups an ANCOVA was performed. Receiver operating characteristic (ROC) curves were calculated to evaluate whether change in knee gait biomechanics could be used to correctly classify patients into either the good or poor outcome group. The area under the ROC curve (AUC) and 95% confidence intervals (CI) were calculated. An AUC of at least 0.70 was considered to be appropriate [25]. Differences in baseline data and change in VAS pain raw scores between the good and the poor outcome group were evaluated using independent samples t-tests and Mann Whitney *U* test, depending on data distribution. Fisher’s exact test was used to determine whether the proportion of patients differed between the groups with regards to the KL classification of radiographic severity of osteoarthritis.

## Results

### Preoperative differences between groups

**Fig. 2:**
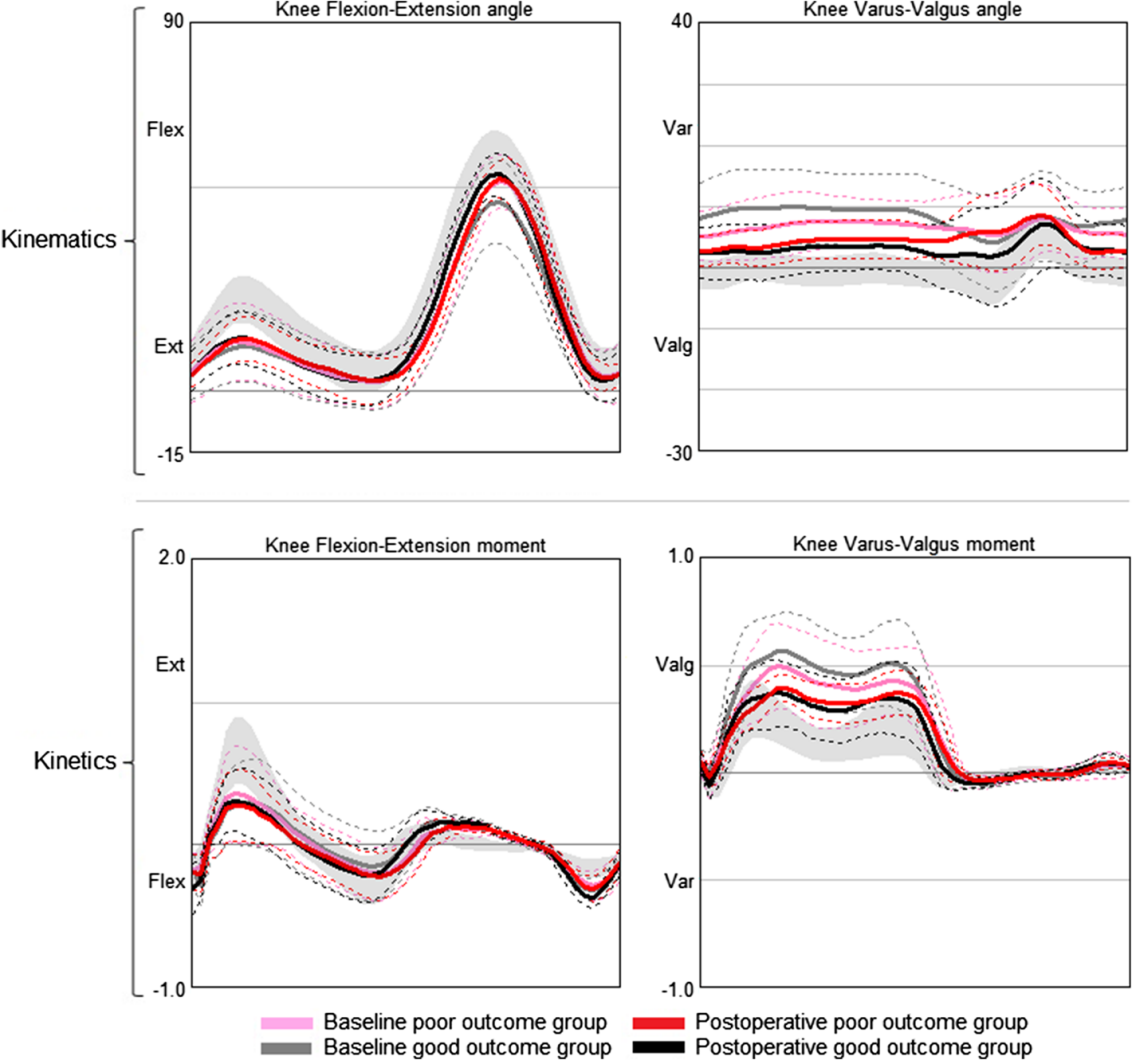
Knee kinematics (degrees) and kinetics (Nm/kg) at baseline and at one year follow-up. Patients are grouped according to postoperative change in knee-related Quality of Life; the good outcome group (*n* = 19), the poor outcome group (*n* = 9). The solid lines represents the mean for each group, respectively. The dashed lines represent ± 1 standard deviation for each group, respectively. The shaded area represents the mean ± 1 standard deviation of an age matched healthy control group (*n* = 25)

The good outcome group presented with significantly less knee flexion-extension range (5°) during gait preoperatively (Fig. 2), compared to the poor outcome group (*p* = 0.004) (Table 2). The proportion of patients with less radiographic changes (a KL score of 3b) were larger in the poor outcome group, although not statistically different (*p* = 0.08) (Table 1). Preoperatively, the poor outcome group reported significantly lower scores in the KOOS ADL subscale (Table 1). In both the good and the poor outcome groups several patients had gone through previous knee arthroscopy. These arthroscopic surgeries were done for diagnostic purposes for some, and in some cases it was due to degenerative meniscal tears (the tears were not repaired, only resected). The number of participants with a previous history of knee arthroscopy were equally distributed in the two groups (11 out of 19 in the good outcome group (57%), and 5 out of 9 in the poor outcome group (56%).

**Table 2:** Passive range of knee motion, knee gait biomechanics, and time and distance parameters at baseline and one year after total knee arthroplasty. Patients are grouped according postoperative change in knee-related quality of life

	Control group ***n*** = 25	Good outcome group, *n* = 19		Poor outcome group, *n* = 9		Effect of interactions	Within group differences	Between group differences	Postop ∆ adj. for baseline
	Reference	Baseline	Postoperative	Baseline	Postoperative		*p*	*p*	*p*
Passive range of knee motion, degrees									
Flexion, mean (SD)	136 * **‖** (5)	119 (15)	115 (10)	124 (16)	112 (14)	0.182	a) 0.306 ***b) 0.025***	c) 0.375 d) 0.516	-
Extension, mean (SD)	2 (4)	−5 (7)	0 (3.5)	0 (7)	0 (6)	0.152	***a) 0.007*** b) 0.814	c) 0.187 d) 0.880	-
Knee kinematics during gait, degrees									
Peak flexion, mean (SD)	54 * **‖** (4)	48 (10)	53 (6)	53 (6)	52 (4)	0.081	***a) 0.004*** b) 0.973	c) 0.175 d) 0.782	-
Flex-Ext range, mean (SD)	57 **‖** (5)	45 (6)	53 (5)	52 (5)	50 (6)	***0.003***	***a) 0.000*** b) 0.471	***c) 0.009*** d) 0.275	e) 0.143
Peak varus angle during stance, mean (SD)	3.2 * (3.3)	10.5 (6.1)	5.7 (4.8)	8.5 (4.3)	5.6 (2.4)	0.246	***a) 0.000*** ***b) 0.042***	c) 0.375 d) 0.953	-
Knee kinetics during gait, Nm/kg									
Peak flexion moment, mean (SD)	−0.54 **‖** (0.17)	−0.41 (0.11)	−0.49 (0.15)	−0.41 (0.10)	−0.38 (0.11)	***0.044***	***a) 0.009*** b) 0.540	c) 0.966 d) 0.054	-
Peak extension moment, mean (SD)	0.50 * **‖** (0.10)	0.43 (0.19)	0.37 (0.15)	0.39 (0.29)	0.33 (0.21)	0.976	a) 0.171 b) 0.324	c) 0.672 d) 0.555	-
Peak valgus moment, mean (SD)	0.60 * **‖** (0.10)	0.60 (0.18)	0.44 (0.16)	0.54 (0.18)	0.46 (0.06)	0.154	***a) 0.000*** b) 0.069	c) 0.428 d) 0.797	-
Time and distance parameters									
Walking speed, m/s, mean (SD)	1.3 **‖** (0.2)	1.1 (0.2)	1.2 (0.2)	1.1 (0.2)	1.1 (0.2)	0.288	***a) 0.022*** b) 0.721	c) 0.593 d) 0.086	-
Stance phase, % of gait cycle, mean (SD)	60.3 **‖** (1.0)	62.5 (2.6)	61.3 (1.8)	62 (1.8)	61.7 (1.6)	0.184	***a) 0.005*** b) 0.642	c) 0.603 d) 0.484	-

a) *p*-value indicating differences between preoperative and postoperative assessment within the **good outcome group**

b) *p*-value indicating differences between preoperative and postoperative assessment within the **poor outcome group**

c) *p*-value indicating differences between the good and the poor outcome group **preoperatively**

d) *p*-value indicating differences between the good and the poor outcome group **postoperatively**

e) *p*-value indicating **postoperative** differences between groups **adjusted for preoperative values**

* indicating a significant difference (*p* < 0.05) between control group and the **good outcome group postoperatively**

**‖** indicating a significant difference (*p* < 0.05) between control group and the **poor outcome group postoperatively**

### Change in knee biomechanics within the good outcome group

The good outcome group displayed significant improvements in the majority of knee gait biomechanics variables (Fig. 2). During gait, peak knee flexion angle increased by 5°, knee flexion-extension range increased by 8°, peak varus angle was reduced by 4.8°, peak flexion moment increased by 0.08 Nm/kg, and peak valgus moment was reduced by 0.16 Nm/kg (Table 2). The good outcome group also displayed a significant increase in walking speed, a reduction (normalization) of stance phase duration (% of gait cycle), and increased passive range of knee extension by 5° (Table 2).

### Change in knee biomechanics within the poor outcome group

The poor outcome group displayed a significant reduction in peak varus angle during stance phase by 2.9° (*p* = 0.042) (Table 2). Aside from that, no other knee gait biomechanics outcomes, or passive knee joint range of motion showed any significant change one year after surgery (Table 2). Baseline data and postoperative results, for each individual classified as having a poor outcome, are presented separately (Table 3).

**Table 3:** Passive range of knee motion and knee biomechanics during gait at baseline and at one year following total knee arthroplasty among patients reporting a poor outcome in knee-related quality of life

	Passive range of motion				Knee biomechanics during gait									
	Knee flexion		Knee extension		Flexion-extension range		Peak varus angle during stance		Peak flexion moment		Peak extension moment		Peak valgus moment	
	*Degrees*				*Degrees*				*Nm/kg*					
ID	Baseline	Postop	Baseline	Postop	Baseline	Postop	Baseline	Postop	Baseline	Postop	Baseline	Postop	Baseline	Postop
1	120	110	−15	0	49.2	51.7	1.5	2.2	−0.42	−0.47	0.23	0.50	0.32	0.48
2	140	125	5	5	56.2	54.5	12.2	8.9	−0.46	−0.41	0.31	0.24	0.51	0.48
3	115	105	10	0	48.5	53.8	4.7	2.2	−0.51	−0.54	0.22	0.23	0.40	0.40
4	120	100	0	−5	50.9	55.9	7.9	7.1	−0.44	−0.39	1.11	0.79	0.57	0.52
5	140	130	5	5	55.4	54.9	16.0	6.3	−0.43	−0.36	0.21	0.42	0.84	0.45
6	90	115	−5	−5	55.0	50.0	7.4	6.4	−0.37	−0.33	0.17	0.21	0.26	0.35
7	130	105	0	5	50.9	39.8	6.2	7.7	−0.39	−0.43	0.49	0.13	0.60	0.42
8	130	90	−5	−10	58.7	49.1	10.8	3.7	−0.48	−0.32	0.32	0.17	0.65	0.56
9	135	130	0	5	42.1	41.5	9.9	5.8	−0.16	−0.15	0.44	0.30	0.68	0.47

### Postoperative differences between groups

At the postoperative evaluation, there were no differences between the good and the poor outcome groups, other than a tendency for the good outcome group to demonstrate larger increases in peak flexion moment during stance phase (*p* = 0.054) (Table 2).

### Differences in change in pain between the groups

No differences were found between the groups with regards to change in perceived pain during gait trials assessed with VAS, or in perceived pain during gait trials at the postoperative assessment (Table 4).

**Table 4:** Perceived pain during gait trials among patients with knee osteoarthritis preoperatively, one year after total knee arthroplasty, and differences in change between groups. Patients are grouped according postoperative change in knee-related quality of life

	Good outcome group, *n* = 19	Poor outcome group, *n* = 9	Difference between groups, p-value
Perceived pain during gait trials, 0-100			
Preoperative VAS, median (range)	35 (4-79)	45 (4-74)	0.09
Postoperative VAS, median (range)	0 (0-18)	1 (0-50)	0.188
Change in VAS, median (range)	−30 (-4 - -79)	−42 (5 - -72)	0.455

*VAS* visual analog scale

### Predictive value of change in knee gait biomechanics on knee-related QoL post TKA

**Fig. 3:**
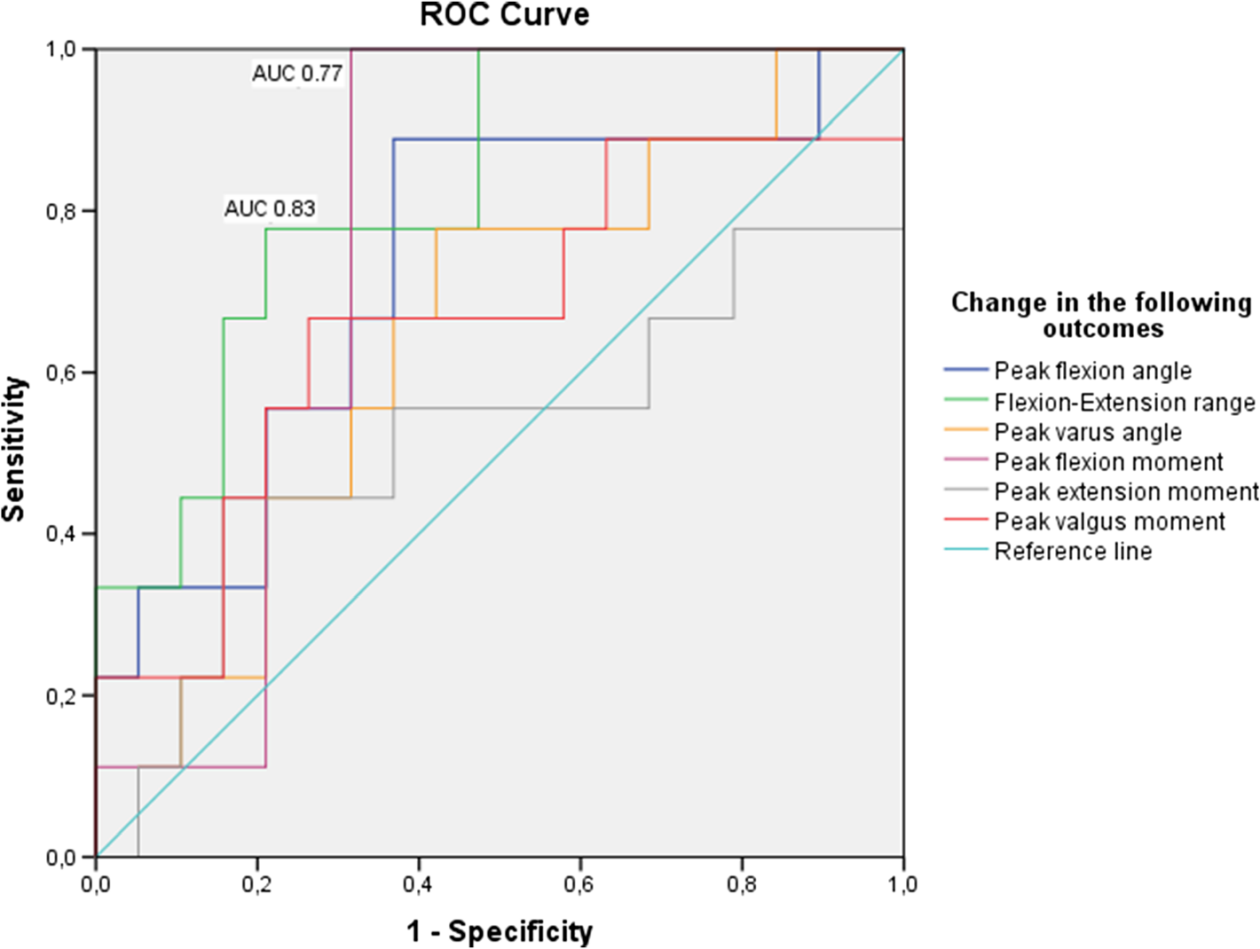
Receiver operating characteristic (ROC) curves were calculated to evaluate the ability of change in knee gait biomechanics to correctly classify patients into either the good or poor outcome group. Poor outcome was defined as change below the minimally detectable change (21.1) in knee-related Quality of Life of KOOS

Receiver operating characteristic (ROC) curves showed that smaller change in flexion-extension range, and change in peak flexion moment had a good ability to predict a poor outcome post TKA (Fig. 3). The AUC was 0.83 for change in flexion-extension range (CI 0.67 – 0.98), and 0.77 for change in peak flexion moment (CI 0.59 – 0.94). The ability to predict a poor outcome in knee related QoL for the other evaluated knee gait biomechanics outcomes were low (Fig. 3).

### Postoperative differences between TKA patients and control group

At the one year follow-up, both the good and the poor outcome groups presented with significantly lower passive range of knee flexion, lower peak flexion angle during gait, lower peak extension moment, and lower peak valgus moment compared to controls (Table 2). The good outcome group was comparable to the control group with regards to walking speed and stance phase duration, while the poor outcome group walked with a reduced walking speed and with a longer stance phase duration as compared to controls (Table 2). The poor outcome group had a significantly lower flexion-extension range, and lower peak flexion moment compared to the control group (Table 2).

## Discussion

This study evaluated change in knee biomechanics during gait among TKA patients classified as having a good or a poor outcome based on postoperative change in knee-related QoL. We found that patients reporting a good outcome in knee-related QoL one year following TKA displayed significant improvements in most knee biomechanical outcomes during gait. Whereas, the only change found at one year after surgery for patients classified as having a poor outcome was a significant reduction in peak varus angle. Even though the sample of patients classified as having a poor outcome was small, data indicate that these patients, who had less severe progression of OA, remained unchanged in knee biomechanics after surgery.

Preoperatively, the good outcome group presented with significantly smaller knee flexion-extension range during gait compared to the poor outcome group. Thus, the good outcome group had a larger potential to improve in knee flexion-extension range after surgery, which they did. The good outcome group displayed significant improvements in most knee biomechanics outcomes during gait, however, these improvements were not necessarily clinically relevant. The increase in flexion-extension range of 8° in the good outcome group seems to be clinically relevant, as the ROC curve analysis showed that a smaller change in flexion-extension range was indicative of a poor outcome in knee-related QoL. In the good outcome group, we also found increased passive range of knee extension and increased knee flexion moment, which we interpret as improved ability to load the knee joint during the latter part of the stance phase. We interpret this increased knee flexion moment as a reflection of patients’ improved confidence in knee joint function, reduced pain, and possibly improved muscle strength as pain has subsided and patients are able to be more physically active. These factors are likely captured in their improved knee-related QoL scores [8]. Pasquier et al. found that improvement in passive knee flexion after TKA was greater for knees with low preoperative flexion, in opposition to knees with preoperative flexion at 110° or larger [26]. In the present study, both groups presented with passive knee flexion larger than 110° preoperatively, and after surgery both groups displayed a slight reduction in passive knee flexion, although not statistically changed. Based on our data, it does not appear to be any clear relationship between passive range of motion and dynamic flexion-extension range during gait.

In the poor outcome group, peak varus angle during stance phase was the only biomechanical variable that improved significantly following surgery. The postoperative degree of varus angle during stance phase, in both the good and poor outcome group, is in agreement with findings of others [27]. Orishimo et al. reported the varus angle to decrease at six months after surgery, but to increase again at the one year follow-up [27]. This, even though static radiographs displayed patients were aligned in a more normalized (neutral) position [27]. Prodromos et al. evaluated the predictive value of static knee alignment on dynamic loading of the knee during gait among patients treated with a high tibial osteotomy and reported it to be low [28]. Further, the authors also found recurrent varus alignment among tibial osteotomy patients presenting with a high external knee adduction moment preoperatively [28]. Similar findings have been reported by Rodriguez et al. who found that dynamic loading patterns of the knee could not be determined by static alignment alone [29]. The results from the ROC curve analysis indicate that smaller change in knee flexion-extension range, and in peak flexion moment are a good predictors of a being classified as having poor outcome after TKA surgery. With regards to surgeon-controlled factors, frontal plane alignment may be the biomechanical factor surgery most successfully can address. However, achieving a good outcome in patient-reported knee-related QoL may not be related to improvements in knee alignment in the frontal plane, but rather to dynamic improvements in flexion-extension range.

Conflicting results have been reported for the influence of preoperative factors and their association with a poor outcome after TKA [30, 31]. Baker et al. reported preoperative variables to have a marginal influence on postoperative satisfaction [30], while results from Judge et al. showed that worse preoperative mental health was a predictor of poor patient-reported outcome [31]. Smith et al. reported the frequency and severity of knee pain post TKA to be partially explained by preoperative joint loading patterns during gait [13]. Scott and colleagues reported that patient expectations, poor mental health, and other musculoskeletal pain had an impact on satisfaction, although the largest determinant of satisfaction after TKA was the level of pain reduction [3]. Using a VAS to monitor perceived pain during gait trials, we found no differences in perceived pain between the good and the poor outcome group. These data suggest pain may not be the primary reason for lower knee-related QoL in the poor outcome group, and that VAS is not sensitive enough to differentiate between groups. Knoop et al. identified five different “clinical” phenotypes in patients with knee osteoarthritis [32], where clinical outcomes differed among these phenotypes. The authors suggest interventions may need to be adapted to these clinical phenotypes [32]. Due to the small samples in the present study, it is not meaningful to classify patients according to these phenotypes, although if we were to speculate, we would expect there to be some individuals in the poor outcome group that fit the description of the phenotype called “minimal joint disease”. It is possible that these individuals may not have had severe joint damage but had pain. Thus, beyond pain relief they did not view the surgery as impacting their knee-related QoL. Future studies should explore whether biomechanical response to knee arthroplasty is different across different clinical phenotypes. It would also be of importance to assess muscle strength pre- and postoperatively, as well as closely monitoring compliance to postoperative rehabilitation.

The number of patients classified as having a poor outcome was higher than expected, compared to previous studies reporting proportions of around 20% of patients with poor outcomes [1–3]. Using the MDC of knee-related QoL as a cut-off for a good or poor outcome, we found a larger percentage of patients with a poor outcome (32%) within our sample. This result may be related to the use of the knee-related QoL subscale which may be a more sensitive measure at one year after surgery, as this is the subscale reported that best demonstrates the improvement occurring between 6 and 12 months [8]. As pain reduction occurs earlier in the postoperative phase, the knee-related QoL subscale is able to capture improvements that takes longer time, such as awareness, ability to trust in the knee, and knee-related lifestyle changes [8]. The percentage of dissatisfied patients is reported to be even higher when evaluating the ability to perform activities of daily living as compared to pain outcomes [2], and this may also be reflected in the subscale knee-related QoL. The higher proportion of patients classified as having a poor outcome could possibly be a consequence of chance in this small sample size. If the MDC cut-off level is a too sensitive measure this would actually minimize the differences, as some patients could have been considered having a good outcome if another measure was used. Hence, some patients classified as having a good outcome might actually be included in the poor outcome group, which we believe makes our results more conservative. It is important to point out, that the MDC is a statistical estimate, and should not be confused with a threshold of what is a *minimal important difference* (MID) [33]. According to King, the MID of a patient-reported outcome will likely depend on the baseline values from which the patients starts, and may differ between groups and settings [33].

Limitations of this study includes that it is a secondary analysis of a prospective cohort study, thus, a priori power calculations were conducted with another aim. Post hoc power calculations deemed sufficient for comparisons within the good outcome group, while power was low in the poor outcome group. The sample size of the poor outcome group may predispose the results towards type II error in reporting no significant change, therefore conclusions are limited. Additionally, we did not monitor aspects of the postoperative rehabilitation, other than record the duration, nor did we have data on muscle strength which we also consider to be limitations of the present study. The strengths of this study include the use of objective measures of knee biomechanics during gait combined with the use of reliable, valid and responsive patient-reported instruments for assessing outcome after TKA. The total sample size is consistent with, and even larger than similar studies using 3D gait analysis [27, 34]. Furthermore, data were collected prospectively with an acceptable rate of follow-up. Seven different senior orthopedic surgeons performed the surgeries making the results generalizable to patients with osteoarthritis treated with TKA in the orthopedic community.

## Conclusion

In this prospective cohort study, we evaluated changes in knee biomechanics among patients classified as having either a good or a poor outcome in knee-related QoL one year after TKA. We found that patients classified as having a good outcome in knee-related QoL improved in knee biomechanics during gait, while patients classified as having a poor outcome, despite similar reduction in pain, remained unchanged in knee biomechanics one year after TKA. With regards to surgeon-controlled factors, frontal plane alignment may be the biomechanical factor surgery most successfully can address. However, achieving a good outcome in patient-reported knee-related QoL may not be related to improvements in knee alignment in the frontal plane, but rather to dynamic improvements in knee flexion-extension.

## Abbreviations

3D: Three-dimensional
ADL: Activities of daily living
BMI: Body mass index
KL: Kellgren and lawrence
KOOS: Knee injury and osteoarthritis outcome score
MDC: Minimally detectable change
MID: Minimal important difference
QoL: Quality of life
TKA: Total knee arthroplasty
VAS: Visual analogue scale
